# Components of hospital personnel preparedness to evacuate patients in disasters: a systematic review

**DOI:** 10.1186/s12873-024-00942-x

**Published:** 2024-02-06

**Authors:** Shandiz Moslehi, Mohammad Masbi, Nader Noori, Fereshteh Taheri, Samira Soleimanpour, Sajjad Narimani

**Affiliations:** 1 Health Management and Economics Research Center, Health Management Research Institute, Iran University of Medical Sciences, Tehran, Iran; 2 Associate professor at the Department of Health in Disasters and Emergencies, School of Health Management and Information Sciences, Iran University of Medical Sciences, Tehran, Iran; 3 Student Research Committee, Iran University of Medical Sciences, Tehran, Iran; 4 Medical Librarianship and information sciences, Educational development center (EDC), Iran University of Medical Sciences, Tehran, Iran; 5 Department of Nursing and midwifery, School of nursing, Social Determinant of Health Research Center, Ardabil University of Medical Sciences, Ardabil, Iran; 6 School of Health Management and Information Sciences, No. 6, Rashid Yasemi St. Vali-e Asr Ave, Tehran, Iran

**Keywords:** Hospital personnel, Preparedness, Patient evacuation, Disaster

## Abstract

**Background:**

During natural catastrophes, hospital staff members’ readiness for crisis management—particularly concerning patient evacuation and improving their safety—becomes paramount. This study aimed to identify the components contributing to hospital staff members’ preparedness to evacuate patients in an emergency.

**Method:**

A systematic review was conducted by searching databases such as Scopus, Web of Science, PubMed, ProQuest, and grey literature through May 2023. Studies that offered unique qualitative or quantitative data regarding hospital personnel readiness to evacuate patients in an emergency were included. Thematic analysis and descriptive statistics were used to examine the extracted data points.

**Results:**

In total, there were 274 scientific articles. The total number of unique studies decreased to 181 after removing duplicate articles. 28 papers that were deemed appropriate for additional study were found based on the titles and abstracts of these articles. Eighteen papers that met the inclusion criteria were selected for the systematic review after their entire texts were finally assessed. Hospital staff preparedness for patient evacuation was divided into four primary topics and nineteen sub-themes. The four primary themes that emerged were management, communication, individual issues, and training on the evacuation process.

**Conclusion:**

The implementation of proper disaster evacuation training programs can be achieved by elevating the perceived sensitivity and protective motive of personnel and considering the personnel’s stages of change. Training hospital staff to properly evacuate patients during disasters is also significantly impacted by other factors, such as effective administration, leadership and prompt and efficient communication.

## Introduction

A disaster refers to any unexpected and sudden event that leads to the weakening and destruction of economic, social, and physical capabilities, resulting in life and financial losses, as well as the destruction of infrastructure facilities, economic resources, and employment opportunities within society [1–3]. While some disasters such as floods and hurricanes can be predicted to some extent, earthquakes remain less predictable [4, 5]. Consequently, disaster preparedness becomes a fundamental principle in crisis management for any country, with a particular emphasis on safeguarding critical facilities like hospitals and their staff during such critical times [6]. Developing and implementing a preparedness plan is essential for effective disaster management [7].

Planning for disasters is an interdisciplinary activity that should be handled by the disaster management body at the national level with thorough local and macro country evaluations, particularly in nations that are more prone to disasters. A grand plan can consider the local economic, social, political, and cultural circumstances in addition to the individual staff members’ motives [8].

Everyone wants to survive a crisis, but hospital staff members have an additional responsibility to save patients while ensuring their safety. This ability needs robust mental and physical preparedness as well as great motivation to carry out evacuation [9, 10]. 

Hospitals should carry out their mission of serving patients and providing care even in a calamity. As a result, the hospital’s technical managers must carry out the necessary and sufficient management for these centers to remain strong, and the staff members who are directly involved in providing care need to have a particular plan in place to get ready for emergencies so that they can tend to the needs of hospitalized patients and handle a high volume of injured patients who come to the hospital [11].

Hospital personnel preparedness for disaster management, specifically in patient evacuation and enhancing their safety, becomes a priority during natural disasters. Analyzing the knowledge and readiness level of hospital personnel concerning this issue can significantly impact the vulnerability or safety of patients during evacuation in the face of natural hazards [12].

Natural disasters like earthquakes, large storms, wildfires and man-made disasters can potentially damage crucial hospitals. Ensuring safe evacuation under such conditions necessitates a well-defined command structure, appropriate equipment, situational awareness measures, local coordination, and high resilience [13]. Due to the unpredictable nature of disasters, ongoing disaster preparedness and training are imperative to enhance the ability to respond to such events and safely evacuate critically ill patients. Hospital personnel play a crucial role in disaster preparedness, but many may need more confidence to respond effectively and understand their roles during these occurrences. When forced evacuations occur, healthcare providers are held accountable as victims and responsible authorities. Hence, training personnel to comprehend their roles during natural disasters is paramount [14].

Studies have shown that increasing personnel training and education in disaster scenarios correlates with an increase in hospital personnel’s confidence to evacuate patients during such events. Utilizing simulations replicating hazardous conditions and placing personnel in such scenarios has proven highly successful in augmenting disaster management and patient evacuation preparedness [15, 16].

To plan for more excellent hospital staff preparation to carry out evacuation operations in disaster conditions, this study aimed to identify the components of hospital personnel’s readiness to evacuate patients in such circumstances.

## Methods

This study was carried out using theme content analysis and systematic review methodologies. The Preferred Reporting Items for Systematic Reviews and Meta-Analyses (PRISMA) guidelines were adhered to by the systematic review [17]. At first, the search strategy was defined based on PRISMA to search, screen, select, and extract data from the studies. This step and quality evaluation of the studies were done by six researchers independently. The following research phase was a thematic content analysis of the data, including extracting the initial codes, defining themes, and providing the draft.

### Eligibility criteria

This research included any published articles (original, review, communication, editorial, opinion, etc.) addressing the following keywords: Evacuation, Hospital, Healthcare workers/ personnel, Preparedness, and Disasters. The exclusion criteria were: (1) articles that did not focus on preparing hospital personnel to evacuate patients in disasters; (2) non-English articles.

### Ethical approval

The Iran University of Medical Sciences’ Ethic Committee granted ethical permission. (IR.IUMS.REC.1402.299).

### Search strategy

The abovementioned keywords were searched on four primary databases: PubMed, Web of Science, Scopus, ProQuest and Google Scholar web search engine. The search was concluded on 2023 May 5 (See Table 1).

**Table 1:** The search strategy of the study

Database	Query	Number of paper
PubMed	(“Health care provider*” [Title/Abstract] OR “Health Personnel” [MH] OR “Health Personnel” [Title/Abstract] OR “Healthcare Worker*”[Title/Abstract] OR “Health Care Professional*”[Title/Abstract]) AND (Evacuat*[Title/Abstract]) AND (Disasters[MH] OR Disaster*[Title/Abstract])	155
Web of Science	TS=(“health care provider*” OR “Nursing preparedness” OR “Nursing Education” OR “Health Personnel” OR “Healthcare Worker*” OR “Health Care Professional*”) AND TS=(Evacuat*) AND TS=(Disaster*)	35
Scopus	TITLE-ABS-KEY (“health care provider*” OR “Nursing preparedness” OR “Nursing Education” OR “Health Personnel” OR “Healthcare Worker*” OR “Health Care Professional*”) AND TITLE-ABS-KEY (Evacuat*) AND TITLE-ABS-KEY (Disaster*)	78
ProQuest	Ab, Ti(“health care provider*” OR “Nursing preparedness” OR “Nursing Education” OR “Health Personnel” OR “Healthcare Worker*” OR “Health Care Professional*”) AND Ab, Ti(Evacuat*) AND Ab, Ti(Disaster*)	6

### Quality assessment of included studies

The records resulting from the searches were imported to EndNote v.20 for Windows. After excluding duplicate records and the articles that had the research exclusion criteria, the title/abstract of the final records was screened to identify relevant articles. Then, a full screening of the remaining articles was conducted.

Two members of the university’s Department of Health in Disasters and Emergencies initially assessed the quality of the incoming articles using the STORB, JBI, and CASB checklists. Two more group members then gave it another look, and finally, the articles were examined in more detail using thematic content analysis.

### Data screening process

Thematic analysis and descriptive statistics were used to examine the extracted data points. Descriptive analyses were used to categorize each article based on authors, topic, type of study, year, country and conclusion. Also, the thematic content analysis approach was adopted to determine the preparedness components of hospital personnel for the evacuation of patients.

## Results

**Fig. 1:**
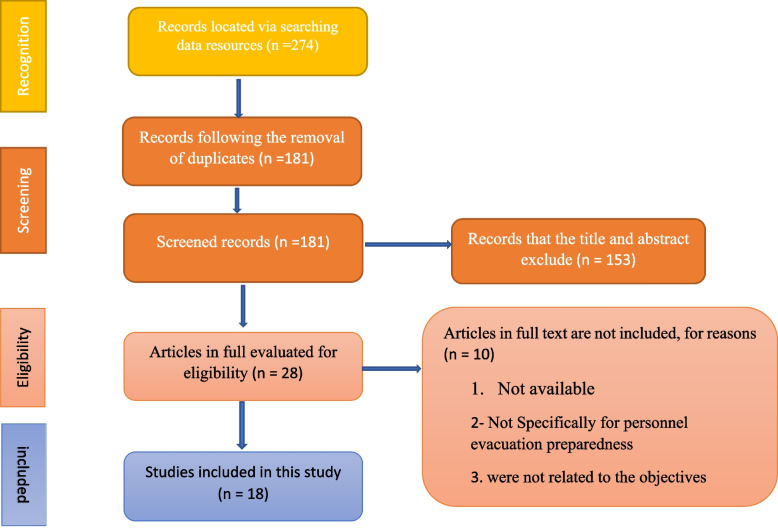
PRISMA (Version: 2020) Flowchart of a systematic review on the preparedness components of hospital personnel for the evacuation of patients in disasters

In the initial electronic search across various databases (PubMed, ProQuest, Web of Science, Scopus), a total of 274 scientific articles were found. After eliminating duplicate articles, the total number of unique studies was reduced to 181 articles. 28 papers that were deemed appropriate for additional study were found based on the titles and abstracts of these articles. Among the 28 articles, 4 were unavailable, and 6 articles were excluded after assessing their full texts. Finally, 18 papers were chosen for the systematic review after meeting the inclusion criteria (See Fig. 1).

### Descriptive statistics

The final selection of studies included those conducted between 2005 and 2020. The selected studies comprised 10 from America, one from Sweden, one from Spain, one from Australia, one from New Zealand, two from China, and two from Japan. The characteristics of the selected studies are shown in Table 2.

**Table 2:** The characteristics of the retrieved papers in a systematic review of hospital staff members’ readiness for patient evacuation during emergencies

N	Authors	Goals	Type of Study	Year	country	Conclusion	Checklist
[1]	Mary A et al.	Assessing caregivers’ attitudes toward systems, tools, and evacuation readiness as well as their readiness to evacuate critical care units both before and during a disaster.	cross-sectional	2015	USA	ICU providers could benefit from being included in hospital evacuation plans and training in future critical care evacuation planning, the creation of standardized tools and procedures for evacuation communication, and the collection of a common dataset from all hospitals that are evacuating.	STROBE
[2]	Megan M et al.	To inform the learning objectives of training programs for reacting to emergencies and safely evacuating patients, evidence-based knowledge is required…	Angoff method (Expert panel)	2019	USA	Decision balance, communicating in hazardous situations, and anticipating future issues should all be covered in advanced nursing education to ensure that charge and resource nurses can support bedside nurses during evacuation events.	CASP
[3]	Megan M et al.	To prepare pediatric nurses for the evacuation of a sick baby from the neonatal intensive care unit (NICU) and pediatric emergency department (ED), a comparison between live evacuation simulations and prerecorded narrated multimedia lectures was conducted.	randomized control group design using post-training clinical simulation	2020	USA	When compared to multimedia lectures, virtual simulation was well-received by nurses and could be a useful addition to nursing education on the evacuation of infant patients in the event of a crisis.	JBI
[4]	Sarah B et al.	(1) Examine the experiences and reactions of personnel intending to shelter, fragile Hurricane Katrina evacuees; and (2) Determine ways to improve planning and training for personnel who might assist elderly, frail patients during and after catastrophes.	semi-structured interviews	2005	USA	Providing residents and evacuees with emotional assistance in addition to physical care during and after disasters should be emphasized in long-term care staff training.	CASB
[5]	Erika L et al.	There are currently highly robust ICU evacuation plans in place for the National Public Hospital System in the event of a fire.	cross-sectional survey- prospective questionnaire-based study	2017	Sweden	More focus is required on identifying the risk associated with unpreparedness. When producing high-quality work in the ICU is the aim, then the general leadership and medical staff readiness for an emergency ICU evacuation should be part of that effort.	STROBE
[6]	Bruno P et al.	Patient-centered planning for a medical facility’s evacuation	cross-sectional survey	2012	USA	The study’s findings can be used by healthcare facilities to project the resources they will need in the event of an emergency evacuation. This will make it possible for the Incident Management Team to assemble the community’s and hospital’s resources needed to guarantee the safest possible patient evacuation.	STROBE
[7]	Parra C et al.	(1) to ascertain whether Spanish PED have documented disaster plans; (2) to delineate the nature of these preparations; and (3) to ascertain whether they encompassed pediatric casualties.	Observational descriptive multicenter study	2012	Spain	The outcome demonstrated the necessity for enhanced catastrophe response preparations.	STROBE
[8]	Schnitker L, et al.	to examine how Australian residential aged care facilities (RACFs) prepared for natural disasters, with a particular emphasis on issues that affected dementia patients.	cross-sectional	2019	Australian	The findings demonstrated that facilities must have sufficient procedures and plans in place to reduce the likelihood of natural disasters.	STROBE
[9]	Thomas A, et al.	Transporting the patient safely and efficiently is the main objective of pediatric patient evacuation.	Intervention/simulation	2020	USA	The findings indicated that Nurses felt more equipped to properly evacuate a seriously ill	JBI
[10]	VanDevanter N, et al.	To investigate the obstacles and resources faced by nurses in performing their duties, as well as the implications for nursing education and disaster preparedness.	cross-sectional	2017	USA	More practical disaster planning activities should be incorporated into disaster preparedness curriculum in nursing schools and practice settings.	STROBE
[11]	Paul H. L et al.	To assess the efficacy of an online course on fire safety and evacuation for healthcare professionals in China.	randomized controlled trial using convenience sampling	2018	China	Healthcare personnel’s understanding of fire safety and evacuation can be significantly increased by an online fire training program that is offered via instructional videos.	JBI
[12]	Xu L et al.	To outline the adverse events (AE) that patients under the unified government organization experienced during the Yushu earthquake, evaluate the operation’s achievements, and provide a comprehensive knowledge of its shortcomings.	The study was an observational, retrospective investigation	2013	China	After significant disasters, a large number of patients can be safely and effectively evacuated by Aeromedical vehicles. However, issues like a shortage of appropriate Aeromedical vehicles and medical supplies, along with a lack of professional medical training in patient evacuation, were made evident following the Yushu earthquake.	STORBE
[13]	Hiroko M et al.	To understand the effects of PHNs’ independent work as well as the various roles they play in evacuation shelters over time following a disaster.	semi-structured interviews	2020	Japan	Allocating human resources is a crucial part of providing post-disaster support to community members.	CASB
[14]	Gloria G et al.	To clarify the shared interpretations that the perinatal nurses had of their experiences as nurses during Hurricane Katrina when they were delivering treatment in the New Orleans area	Qualitative (Semi-structured, in-depth interview)	2008	USA	The key assets include a strong foundation in basic nursing, natural problem resolution, and a feeling of cohesion among staff members. Continuous supportive interventions are necessary for nurses and other caregivers to recover from and manage symptoms related to exposure to trauma.	CASB
[15]	Kelly R et al.	To compile the evidence that currently exists indicating how adequately prepared nurses are for disasters in developing nations.	descriptive	2007	USA	The nursing profession must recognize its importance in the catastrophe domain and take on leadership positions constantly as the number of disasters rises.	STROBE
[16]	Claire P. et al.	This study examined the experiences of the evacuees and found lessons that could be applied to disaster preparation in the future.	Qualitative study	2013	New Zealand	Older persons expressed anxiety, and caregivers reported cognitive issues. Communication issues were a big worry.	CASB
[17]	Timothy P. et al.	To learn more about the experiences of Veterans Health Administration (VHA) professionals and veterans with SCI/D concerning recent natural disasters, as well as to pinpoint lessons learned for SCI/D-related disaster preparedness and response.	Qualitative study Semi-structured interview	2011	USA	During disasters, patients with spinal cord injuries and their healthcare professionals encounter urgent requirements even with good planning, so it’s important to figure out how to coordinate care. Lessons gained are meant to alert medical professionals who treat patients with spinal cord injuries or disorders about potential future hazards.	CASB
[18]	Yuko K et al.	We examined how our hospital operated during the first ten days following the Great East Japan Earthquake and the Fukushima Daiichi nuclear power plant accident.	Quantitative	2014	Japan	Future hospital planning for nuclear catastrophes should account for the scarcity of personnel and medical supplies required to sustain at least five days of isolation.	STROBE

### Thematic content analysis

Four major topics and 19 sub-themes were identified from the systematic review and thematic content analysis that describe hospital staff members’ readiness for patient evacuation during a crisis. The main themes included evacuation process training, individual factors, communication and management. The sub-themes included educational approach, Target groups of education, educational content, physical and psychological aspects, spirituality, knowledge, disaster experience and skills, ethical issues and considerations, ethical issues and considerations, internal and external organizational communications, healthcare staff-patient communication, doing collaborative work, having Communication equipment, Participation of the patient’s family in the evacuation process, managing patients during evacuation, evacuation planning, leadership and decision-making process, existing standards, rules and policies for evacuation, and resource allocation and administration (See Table 3).

**Table 3:** The elements of hospital staff readiness for patient evacuation during emergencies, as determined by theme content analysis and systematic review

Evacuation process training	Educational approach	Gamification [18] Practice [18–24] Workshop [20] simulation [19, 23, 25] speech method [18, 22] Online training [18, 22]	
	Target groups of education	patients [23, 24] General population [20] Vulnerable group [20] Staff [20, 23, 26]	
	Educational content	Health issues	Environmental Health [27] Prevention methods [22, 27]
		Safety issues	Personal protection measures [27] Disaster awareness [20] Disasters and emergencies Preparedness [23]
***Individual factors***	Physical factors	Physical fatigue [27] long shift [27] Physical ability [26, 28]	
	Psychological factors	Adaptability and mental resilience [22, 24, 29] Anxiety management abilities [23]	
	Spiritual factors	sacrifice [29] religious beliefs [22]	
	Knowledge, disaster experience and skill	Acquaintance and recognition of employees from the evacuation plan [21] Education level of personnel [26, 30] Previous evacuation experience [19, 22, 24, 27, 28] Healthcare proficiency [29–31] Personnel mastery of their duties [32] Having problem-solving skills [29]	
	Ethical issues and considerations	Job and organizational commitment [29] Responsibility [29] Professional ethics [29] Emotional support for patients [33]	
***Communications***	Internal and external organizational communications [18, 23, 24, 29, 32, 34] Healthcare staff -patient communication [27] Doing collaborative work [18, 22, 24, 29] Having Communication equipment [22, 27] Participation of the patient’s family in the evacuation process [25]		
***Management***	Managing patients during evacuation	Correct transfer of patients [22, 24, 25, 33] Preparing patients for evacuation [18, 27] Medical document management [22, 23, 33, 34] Managing vulnerable patients [23, 24, 27, 31, 32] System for patient relocation to other healthcare facilities [21, 33, 34] Therapeutic and palliative care [33, 34]	
	Evacuation planning	Employees Performance appraisal and retraining program [26] Evacuation prioritization [33] Evacuation action plan [21, 22, 24, 25, 29, 33–35] Comprehensive plan of critical evaluation [35] Review the evacuation plan [21] Existence of educational programs [21, 29]	
	Leadership and decision-making process	Department coordination and cooperation [30, 35] Human resources management [18, 19, 22, 33] Timely decision making [32] Personnel participation in decision-making [32]	
	Existing standards, rules and policies for evacuation	The exit route is clear [25] Program uniformity and standardization [32] Early warning system [24]	
	Resource allocation and administration	Human resources	Welfare services and social support [22, 23, 25] The existence of sufficient manpower [18, 27, 33] The existence of trained manpower [25]
		Equipment resources	Efficient resource utilization [31] Having enough equipment [19, 22, 24, 27, 30, 32–35]
		Physical resources and infrastructure	Designated place for evacuation [25] Create a workspace [33]

## Discussion

Evacuation and relocation of hospitals in disasters is one of the necessary and essential indicators of hospital preparedness against disasters [36]. Hospital evacuations are uncommon, but they have complicated mechanisms and numerous repercussions because of the many patients and scarce resources [37]. Considering the importance of the subject, the present study was conducted to examine the components of hospital personnel preparedness to evacuate patients in disasters with a systematic and thematic content analysis method. The results of thematic content analysis led to the extraction of four main themes: education, individual factors, communication, and management.

At a glance, descriptive results show that most of the articles were conducted in America and some were conducted in the continents of Asia, Europe and Australia. This result clearly shows the research gap in this field, especially for areas with natural disasters in the world. America, which is usually at the top of the countries with natural disasters, has had a significant number of studies. On the other hand, some countries such as India, Indonesia, Philippines, and Iran, which have not been spared from the number and financial damage of disasters, have not conducted a study in this regard [38, 39]. Therefore, one of the primary results of this review is the insufficiency of the number of studies in the field of hospital evacuation in emergencies around the world, especially in disaster-stricken countries.

Hospital evacuation is the temporary transfer of people and property to safe places before, during or after a hazardous event to protect them [40]. Therefore, considering that hospital activities during disasters deal with the lives of patients and personnel, the existence of a standard practical guide for hospital activities, including emergency evacuation, is a requirement [41].

The first theme that emerges in this review is education, which includes Evacuation process training for patients [24, 34], personnel [24, 30, 35], and vulnerable groups [35], While one of the most important groups in the timely action to evacuate the hospital, that is, hospital managers, have been neglected. Education is accepted as one of the main pillars of improving disaster management. Different hospital wards need planning in the field of equipment supply, clarification of jobs, organization of triage team members, and cohesion of the organizational structure before unexpected risks occur [42]. These will only be implemented with the training of managers, including hospital managers, matrons and supervisors. Despite the importance of training in clarifying hospital evacuation processes in disaster situations, studies have yet to be conducted worldwide using educational intervention or RCT in this field [19, 26, 43]. Therefore, one of the significant results of this study is showing the insufficient number of training studies for the target groups to evacuate the hospital in accidents.

The second theme in the thematic analysis was individual factors related to staff preparedness for hospital evacuation. Hospital staff should have medical, psychosocial and social support services to maintain physical and mental health, resolve conflicts and work obligations and perform emergency roles in emergencies. In such circumstances, employees who are mentally or physically affected by emergency conditions should be supported in terms of psychosocial, treatment or continuous rehabilitation [44].

Ethical considerations are among the individual factors of personnel preparedness to evacuate the hospital. In this review, topics such as commitment, responsibility, and patient emotional support were extracted from the reviewed studies. Various studies have emphasized that preserving patients’ privacy and respecting their identity are essential moral values that become more important in emergencies such as disasters [5, 45]. Hospital staff will face ethical challenges. On the one hand, they must save their lives; on the other hand, they have to save patients’ lives. All these critical issues must happen in a short period. This indicates the need to support patients and medical staff and directly raises ethical issues regarding whom to evacuate and who has decision-making power. However, studies showed a significant knowledge gap for emergency triage and related ethical and legal concerns [46]. Prioritizing patients in emergencies, when resources are scarce and there is less time to make decisions, is one of the most challenging ethical issues for staff during hospital evacuation [47]. Due to the issue’s importance, the American Medical Association’s Code of Medical Ethics states that due to the medical staff’s obligation to care for the sick and injured, they must provide emergency medical care during disasters. However, due to these issues, it is necessary to pay attention to ethical issues in hospital personnel in advance for a quick and appropriate response to evacuate the hospital in disasters.

Among the individual factors related to people’s preparedness in a disaster were people’s knowledge, experience and skills. Numerous research studies have demonstrated the relevance and efficacy of individual elements, such as knowledge and behavioral skills, in the performance of hospital staff during emergencies and disasters [48, 49]. Studies have shown that only half of the staff have adequate knowledge and performance in hospital emergency evacuation, which indicates the need to develop appropriate intervention strategies to improve the knowledge and performance of hospital employees in the face of possible disasters [50]. A study in southern Iran found that doctors, nurses, and other hospital staff needed more knowledge about disaster preparedness [36]. Another study also showed that stages of change in individuals play a significant role in learning new behaviors [51].

Communication was the third theme identified in the present thematic analysis. Communications, like other hospital activities, may be affected during disasters. According to the unfavorable conditions, different methods should be used to call the hospital personnel to use them when the disaster occurs [52]. Hospitals should identify potential facilities in their evacuation plan, including facility capabilities and the types and numbers of patients they can potentially accommodate. It can pre-display communication that occurs during a discharge event. If physicians are not aware of their role during evacuation, the operational plan should ensure communication of roles and job duties for physicians [47]. Effective communication is essential for a successful emergency evacuation, both inside the hospital and between it and outside organizations [53]. . Communication and information management are among the most essential components of hospital evacuation management during disasters. Worksheets and checklists that are standard help with efficient communication. Emergency evacuation packs, which can include worksheets, phone numbers, facilities and equipment for transfers, and disposable patient care items, should be made and made available by hospitals [54]. One of the critical issues in the field of disaster is risk communication between the public, patients, hospital staff, managers, and media. In such a situation, a proper notification system should be established during hospital evacuation [55, 56], which was not addressed in the studies reviewed in this systematic review.

The fourth theme in the thematic content analysis was management, which included several subthemes including leadership and decision-making, patient management, resource management, standards, and planning. No single formula or algorithm can be prescribed for deciding how to evacuate a hospital in a disaster, however, at least a plan or instruction for hospital evacuation is necessary according to the conditions. In fact, if the hospital has a prepared emergency operations plan and operates according to the hospital’s incident command, the leader, often a senior hospital administrator, will decide to evacuate together with the hospital’s senior staff [57]. Leaders must ensure that the essential needs of patients are met during emergency evacuation. They must also confirm the patient’s identity and the transfer’s destination and have a document confirming the patient’s evacuation from the hospital [47]. An emergency response team consisting of doctors, nurses, midwives, staff trained by emergency management technicians, paramedics and a trained ambulance driver is essential for evacuations [55]. A study in Iran concluded that the hospital incident command system can effectively increase hospital staff’s readiness and speed of action during disasters [58]. Sawano et al. suggested that hospital evacuation insights during disasters should be considered in disaster preparedness policies, planning, training and pre-testing of these plans [59]. As in other organizations, all aspects of decision-making in hospitals must be implemented and monitored. Establishing the necessary coordination with senior management is essential in making good decisions about hospital evacuations in disasters [53].

The number of studies on the preparedness of hospital personnel to evacuate patients in disasters was few, and the lack of access to the full text of several articles led us to review only 18 relevant articles on this topic.

## Conclusions

In general, the results of this systematic review using 18 studies conducted worldwide showed that evacuation process training, individual factors, communication, and management are the practical components of the preparedness of hospital staff for hospital evacuation in disasters. Without taking into account the individual conditions of the personnel and the stages of change of each personnel and staff protection motivation, the training process will not be effective, so first the stages of change of personnel should be identified and then proper training should be given. It is suggested that more interventional and educational studies be conducted to improve the knowledge and performance of hospital personnel regarding emergency evacuation. To prepare a suitable training program for hospital personnel, it is first necessary to determine the stages of change of health care personnel based on the TTM model. Afterward, it is essential to assess the motivation of employees to assist in patient evacuation according to protection motivation theory. Finally, a suitable training program can be determined by assessing the people’s readiness. There must be much exercise so that the stress of the emergency does not cause forgetting.

## Data Availability

No datasets were generated or analysed during the current study.
